# Glutaraldehyde – A Subtle Tool in the Investigation of Healthy and Pathologic Red Blood Cells

**DOI:** 10.3389/fphys.2019.00514

**Published:** 2019-05-14

**Authors:** Asena Abay, Greta Simionato, Revaz Chachanidze, Anna Bogdanova, Laura Hertz, Paola Bianchi, Emile van den Akker, Marieke von Lindern, Marc Leonetti, Giampaolo Minetti, Christian Wagner, Lars Kaestner

**Affiliations:** ^1^Dynamics of Fluids, Department of Experimental Physics, Saarland University, Saarbrücken, Germany; ^2^Landsteiner Laboratory, Sanquin, Amsterdam, Netherlands; ^3^Theoretical Medicine and Biosciences, Saarland University, Homburg, Germany; ^4^Université Grenoble Alpes, CNRS, Grenoble INP, LRP, Grenoble, France; ^5^Red Blood Cell Research Group, Institute of Veterinary Physiology, Vetsuisse Faculty and the Zurich Center for Integrative Human Physiology (ZIHP), University of Zurich, Zurich, Switzerland; ^6^UOC Ematologia, UOS Fisiopatologia delle Anemie, Fondazione IRCCS Ca’ Granda Ospedale Maggiore Policlinico, Milan, Italy; ^7^Laboratory of Biochemistry, Department of Biology and Biotechnology, University of Pavia, Pavia, Italy; ^8^Physics and Materials Science Research Unit, University of Luxembourg, Luxembourg City, Luxembourg

**Keywords:** glutaraldehyde, erythrocytes, hemolytic anemia, fixation, cell shapes, stiffness, osmolality, batch variation

## Abstract

Glutaraldehyde is a well-known substance used in biomedical research to fix cells. Since hemolytic anemias are often associated with red blood cell shape changes deviating from the biconcave disk shape, conservation of these shapes for imaging in general and 3D-imaging in particular, like confocal microscopy, scanning electron microscopy or scanning probe microscopy is a common desire. Along with the fixation comes an increase in the stiffness of the cells. In the context of red blood cells this increased rigidity is often used to mimic malaria infected red blood cells because they are also stiffer than healthy red blood cells. However, the use of glutaraldehyde is associated with numerous pitfalls: (i) while the increase in rigidity by an application of increasing concentrations of glutaraldehyde is an analog process, the fixation is a rather digital event (all or none); (ii) addition of glutaraldehyde massively changes osmolality in a concentration dependent manner and hence cell shapes can be distorted; (iii) glutaraldehyde batches differ in their properties especially in the ratio of monomers and polymers; (iv) handling pitfalls, like inducing shear artifacts of red blood cell shapes or cell density changes that needs to be considered, e.g., when working with cells in flow; (v) staining glutaraldehyde treated red blood cells need different approaches compared to living cells, for instance, because glutaraldehyde itself induces a strong fluorescence. Within this paper we provide documentation about the subtle use of glutaraldehyde on healthy and pathologic red blood cells and how to deal with or circumvent pitfalls.

## Introduction

Besides its application as disinfectant and medication, glutaraldehyde is used in biomedical research to fix cells. The principle behind the fixation is the binding of glutaraldehyde to nucleophiles of which the amino groups are the most abundant but binding to, e.g., sulfhydryl groups also occurs (Griffiths, 1993). The result is a crosslinking of the proteins of the cell (Kawahara et al., 1997), Figure 1. Approximately 45–50 years ago, glutaraldehyde and its properties were a hot research topic (Richards and Knowles, 1968; Hardy et al., 1969; Robertson and Schultz, 1970; Morel et al., 1971; Gillett and Gull, 1972; Rasmussen and Albrechtsen, 1974; Squier et al., 1976). Such studies mention the abundance of this molecule in monomeric and polymeric form, discussing the formation of polymers at high temperatures and arguing the influence of monomers and polymers in fixation efficiency. Nowadays glutaraldehyde is “only” used as a tool to fix cells. Especially for rare red blood cell (RBC)-related diseases, cell shape is an important diagnostic parameter and fixation is an approach to circumvent the subtle sample transportation challenge (Makhro et al., 2016; Hertz et al., 2017). However, a large variety of protocols exist due to laboratory specific customs and habits. Even for the storage of the glutaraldehyde a non-representative poll among collaborators revealed storage from room temperature, refrigerated to frozen conditions.

**FIGURE 1:**
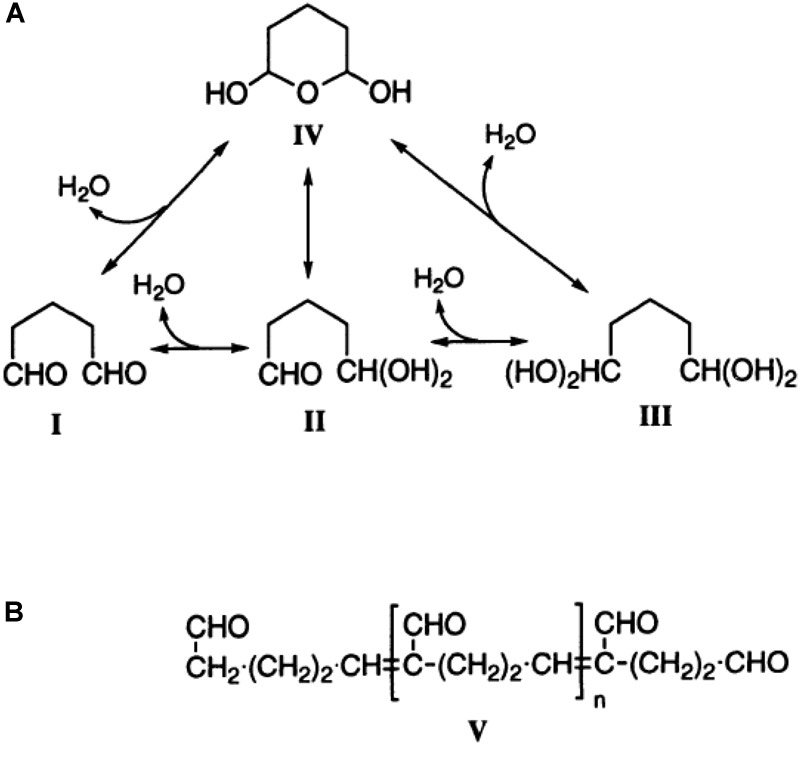
The structure of glutaraldehyde. **(A)** The molecular structure of glutaraldehyde in non-concentrated aqueous solution and the possible conversion paths. **(B)** The principal structure of glutaraldehyde in aqueous solution in the current theory in biochemistry. This figure is a reprint from Kawahara et al. (1997).

Along with the fixation comes an increase in the stiffness of the cells. In the context of RBCs this increased rigidity is often used to mimic malaria infected RBCs because they are also stiffer than healthy RBCs (Aingaran et al., 2012). Furthermore, hydrodynamic studies on blood flow and RBC interactions use glutaraldehyde as a parameter to change cellular rigidity, supposing a gradual concentration dependent process increases the stiffness of the cells.

Here we summarize properties and provide original data of glutaraldehyde’s action on RBCs and stress the parameters that are important in particular in respect to RBC related pathologies to avoid pitfalls and to allow data reproducibility as well as interlaboratory-comparability.

## Materials and Methods

### Blood Collection

Donors and patients were enrolled in the study after signing an informed consent. The procedure is approved by the local ethics committee (approval no. 51/18) and was performed in accordance with the Helsinki international ethical standards on human experimentation. Venous blood was collected into EDTA coated tubes (1.6 mg/ml). RBCs were isolated from the whole blood by washing (centrifugation, 380 × *g*, 5 min) 3 times with PBS (Sigma, Germany).

### Glutaraldehyde Supply

Partly because this paper reflects a collaborative project including partners at different locations and partly by purpose, different sources of glutaraldehyde have been used throughout this study. The source (supplier), the mode of storage and in which experiments/measurements they were used in are summarized in Table 1.

**Table 1:** Sources of glutaraldehyde and in which measurements they were used.

Supplier and grade	Mode of storage	Measurements used
Sigma, grade I, 25% in H_2_O	-20°C; more than one year of storage, thawed for every use	Figure 2A,C; batch 1 in Figure 3B, 4B,C

Merck 25% in H_2_O	Refrigerated	Figure 2B

Sigma, grade I, 25% in H_2_O	-20°C; fresh batch	Figure 3A,C, 5, 6B,D, 7B, 8; batch 2 in Figure 3B, 4B,C; dashed line in Figure 4A; bold lines in Figure 6A

Merck 25% in H_2_O	room temperature for more than one year	batch 3 in Figure 3B, 4B,C; solid line in Figure 4A

Sigma, grade I, 50% in H_2_O	-20°C, aliquoted upon delivery	batch 4 in Figure 4B,C; thin lines in Figure 6A

Fluka 25% in H_2_O	room temperature for more than one year	Figure 7A, 9

### RBC Stability Test

PBS solutions (39.4 ml) with various concentrations of glutaraldehyde (various suppliers) were prepared in 50 ml tubes. Packed RBCs (600 μl) were pipetted slowly into the solution. The large tube and the high solution/cell volume ratio (65:1) was chosen to make sure the sample had sufficient volume to fix as individual cells and would not form aggregates. The tubes were placed on a tube roller for 1 h to allow for glutaraldehyde to fix. After the fixation procedure, glutaraldehyde was removed by washing the cells 3 times with PBS and resuspended in the same solution. To test the “stability” of the cells the supernatant was spectroscopically tested for hemoglobin to identify the portion of lysed cells. For the spectrophotometry each tube was resuspended completely until the whole sample appeared homogenous. Three milliliter from each sample were placed into a new tube and centrifuged at 500 × *g* for 5 min to get a clear distinction between the pellet and the supernatant. One milliliter from the supernatant was placed in a spectrometer cuvette and was diluted 1:3 with PBS to ensure the hemoglobin absorption value is within the limits of the spectrophotometer (Red Tide, Ocean Optics, Netherlands). The hemoglobin absorption peak of the Soret band at about 420 nm was observed and compared between the samples. As a 100% hemolysis reference, healthy RBCs were lysed with distilled water to measure the total hemoglobin content.

### Spectroscopy

To determine the ratio of glutaraldehyde monomers and polymers, UV-absorption spectroscopy was performed at room temperature. The extinction peaks are at 280 nm for monomers and at around 235 nm for polymers (Morel et al., 1971). To determine the monomer-polymer ratio, putative 1% glutaraldehyde samples were prepared in water. Spectra were recorded on these samples for wavelengths from 200 nm to 350 nm on Thermo Scientific Evolution 220 (Thermo Fisher, United States). To measure trypan blue’s absorption spectra, 0.01% trypan blue (Sigma-Aldrich, United States) solution was prepared in PBS and recorded for wavelengths from 200 to 750 nm. The hemoglobin absorption spectrum was measured as detailed before (Kaestner et al., 2006).

The emission and excitation spectra of the glutaraldehyde induced fluorescence was measured with a Jasco FP-6500 spectrofluorometer (Jasco, Germany). RBCs were fixed with 1% glutaraldehyde from different batches for one hour, washed three times in PBS and resuspended in PBS to the concentration of 0.01125% to avoid excessive scattering. For the emission spectra measurements, excitation was set to 450 nm and the fluorescence was recorded in the range from 480 nm to 750 nm. For the excitation spectra, emission was set to 540 nm and the excitation scanned from 350 nm to 500 nm.

### Elongation Index

To compare the mechanical properties of RBCs treated with various concentrations of glutaraldehyde, their elongation index was measured by LoRRca Maxsis (Mechatronics, Netherlands). Samples were treated as outlined above (2.2 RBC stability test). For each case 25 μl of 45% cell suspension in PBS were mixed with 5 ml of polyvinylpyrrolidone buffer (PVP, Mechatronics, Netherlands). The range of set shear was 1 to 30 Pa.

### Atomic Force Spectroscopy

In order to investigate the variation between cells at certain concentrations of glutaraldehyde, atomic force microscopy (AFM) was employed. All measurements were performed in PBS with the JPK Nanowizard 3 (Bruker, Germany) setup coupled with a microscope. Effective Young’s modulus of cells was measured through force-distance curves. The variety of cantilevers of MLCT model (Bruker AFM Probes, United States) with different nominal spring constants as well as different indentation forces were tested in order to adapt measurement conditions for each glutaraldehyde concentration. Prior to the measurements cells were immobilized on the substrate with Cell-Tak (Corning, United States). Force mapping was performed for 3–5 cells of each population on a grid of 32 × 32 points, corresponding to a 10 μm × 10 μm map. Force-distance curves were acquired at the indentation rate of 5 μm/s. Curves were analyzed according to the Hertz model, implemented in the JPK software. The Poisson ratio was set to 0.5.

### Measurement of Osmolality

Glutaraldehyde was added to PBS for osmolality measurements. The osmometer (Type 6, Loser Messtechnik, Germany) was checked for zero display with distilled water prior to each measurement. Glutaraldehyde solutions in PBS with various concentrations were diluted 1:10 to have a 110 μl sample.

### Density Measurements

The densities of glutaraldehyde treated cells were determined by adjusting the density of solutions they were suspended in. In an Eppendorf tube with treated cells, the percentage of OptiPrep (Stemcell Technologies, Canada) was adjusted slowly to match the density of treated cells. To determine the cell density values, tubes with known concentration of OptiPrep were centrifuged at 500 × *g*. In the case where the density of the solution matched the density of the cells, no sedimentation was observed after centrifugation.

### Sample Preparation for Microscopy

Ten microliters of blood were diluted 1 to 10 in PBS and directly added to 1 ml of 1% or 0.1% glutaraldehyde in PBS. Additional samples were prepared in order to study the stability of RBCs shapes within the spherocyte-discocyte-echinocyte scale in fixative solutions. The samples were prepared by suspending 30 μl of whole blood in 1 ml NaCl solutions with different osmolality. A hypotonic solution (131 mosmol/kg H_2_O) was used to form spherocytes, a hypertonic solution (800 mosmol/kg H_2_O) for echinocytes and an isotonic solution (290 mosmol/kg H_2_O) was used for discocytes. After inducing the shape transformations, 20 μl of each cell suspension were fixed in 1 ml of the respective NaCl solutions supplemented with 1% or 0.1% glutaraldehyde. Since the addition of 1% glutaraldehyde increases the osmolality of the solutions, extra samples were fixed in respective NaCl solutions that were diluted in order to keep the same final osmolality after addition of 1% glutaraldehyde. Samples were kept in rotation (Grantbio, United Kingdom) for 1 h at room temperature. Consequently, cells were washed three times by addition of 1 ml of each respective original solution and centrifuged at 735 × *g* for 3 min. Finally, cells were resuspended in 1 ml of their original solution. Brightfield images of both live and fixed cells were acquired with a 50× objective (LU Plan 50×, NA = 0.55) on an inverted microscope (Eclipse TE2000-S, Nikon, Japan).

### Staining Procedures

Eosin-5′-maleimide (EMA) membrane staining was performed on both fresh and glutaraldehyde fixed RBCs. Two microliters of blood were added to 50 μl EMA (5 mg/ml) supplemented with 10 mM CaCl_2_ and incubated at room temperature for 2 hours in rotation (Suemori et al., 2015). Samples were washed 3 times with 1 ml of Tyrode solution, containing in mM: 135 NaCl, 5.4 KCl, 5 glucose, 1 MgCl_2_, 1.5 CaCl_2_ and 10 HEPES, pH 7.4, each time spun at 300 × *g* for 2 min. Cells were resuspended in 1 ml of Tyrode solution and imaged in confocal microscopy with a 100x oil objective (Plan Apo TIRF, NA = 1.49, Nikon, Japan) and an excitation wavelength of 488 nm. Glutaraldehyde induced fluorescence was examined at an excitation wavelength of 647 nm.

Some of the samples were fluorescently labeled for 3D image acquisition with confocal microscopy. Staining of the cell membrane was performed either with CellMask^TM^ Deep Red (Life Technologies, United States) or with PKH67(Sigma-Aldrich, United States). Living cells were stained for comparison with fixed samples. CellMask staining was performed by adding 1 μl of CellMask stock solution (0.5 μg/ml) into the RBCs suspension followed by incubation for 5 min and 3 washes prior to resuspension in 1 ml PBS. For PKH67, 10 μl of packed cells were stained with 0.5 μl PKH67 as recommended by the manufacturer and incubated at room temperature for 5 min. 3 washes followed before resuspension in 1 ml PBS. Living cells were resuspended in PBS supplemented with 0.1% bovine serum albumin.

### Flow Cytometry

Fixed cells were used for immunofluorescence tests performed by flow cytometry on cord blood to measure fetal hemoglobin (HbF) content. Ten microliters of whole cord blood were fixed for 10 min in either 0.05% or 1% glutaraldehyde in PBS. An appropriate staining buffer suggested by the company (BD Biosciences, United States) was used for next steps. After one wash, cells were permeabilized with 0.1% Triton X-100 (Sigma-Aldrich, United States) solution for 10 min. Cells were then washed and incubated with FITC mouse anti-human fetal hemoglobin antibody (BD Biosciences, United States) for 20 min at room temperature. After washes, cells were finally resuspended in 500 μl of staining buffer and 100,000 events were recorded by flow cytometry (Gallios Flow Cytometer, Beckman Coulter, United States). Each stained sample was measured together with its respective unstained fixed sample used as a negative control. Living cells were also analyzed for comparison. Plots were obtained with the software FlowJo V10 (BD Biosciences, United States).

### 3D Imaging

Cells were placed between two glass cover slips (24 mm × 60 mm) with a suspension of beads with a 20 μm diameter used as spacers between the cover slips. A 60× oil objective (CFI Plan Apochromat Lambda 60× Oil, NA = 1.4) of an inverted microscope (Eclipse Ti, Nikon, Japan) was used to image. Cells were scanned using a diode laser emitting at 647 nm (LU-NV Laser Unit, Nikon, Japan) in a *z*-range of 20 μm with steps of 300 nm. A spinning disk-based confocal head (CSU-W1, Yokogawa Electric Corporation, Japan) was used to generate the images. 3D reconstruction was later performed with a Matlab R2017b (MathWorks, United States) routine.

### Examination of Autofluorescence Quenching

To test the quenching of glutaraldehyde autofluorescence, fixed cells were permeabilized with 1 ml 0.1% Triton X-100 for 10 min, washed and resuspended in 1 ml PBS. Two hundred microliter of cell suspension were added to 800 μl of 0.4% trypan blue (Sigma-Aldrich, United States) in PBS. After 15 min of incubation at room temperature, cells were washed twice and imaged as described before.

### Scanning Electron Microscopy (SEM)

Fixed samples in 1% glutaraldehyde solution (Fluka, Germany) were centrifuged on circular glass coverslips (10 mm, Schott, Germany) for 6 min at 40 × *g* in a cytocentrifuge (Cytospin 2 SHANDON, United States). Sample slides were immediately resuspended in PBS to avoid sample drying and washed 3 times to remove any glutaraldehyde residues. Post fixation was done for 30 min in a solution containing 1% osmium tetroxide in PBS, followed by 3 washes in PBS, 5 min each. Samples were dehydrated in increasing ethanol concentrations: 30 min in 70% ethanol, 30 min in 80% ethanol and overnight in 100% ethanol. Cells were dried in a critical point drying (Bal-tec CPD030, United States), mounted on aluminum stubs and sputter coated with 4 nm platinum (Safematic CCU-010, Switzerland). SEM (Zeiss Supra 50 VP, Germany) imaging was performed at 10 kV with a secondary electron detector for a 5000× magnification.

## Results

### Fixation vs. Modulation of Stiffness

The protocols so far published are confusing concerning the application of the glutaraldehyde concentration because they range from 0.0005% to 8% (Morel et al., 1971; Tong and Caldwell, 1995; Guo et al., 2014) and all use the term “to fix cells” upon the application of glutaraldehyde.

**FIGURE 2:**
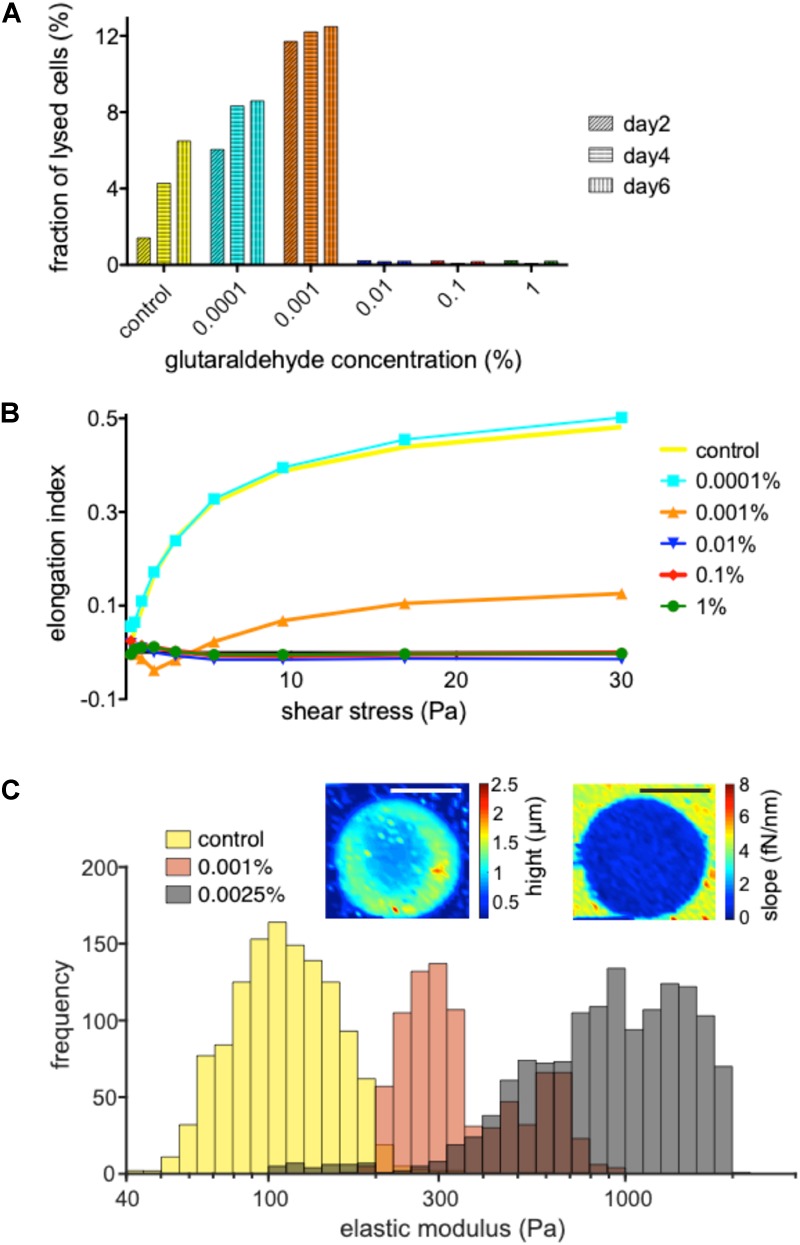
Dependence of fixation and rigidity on the glutaraldehyde concentration. **(A)** Hemolysis of RBC in suspension without and with the specified concentrations of glutaraldehyde 2, 4 and 6 days after withdrawal/initial treatment. Living RBCs (leftmost columns) show an increased hemolysis over time which is further increased with increasing glutaraldehyde concentrations (0.0001 and 0.001%) until the cells reach a fixed state (0.01% to 1% glutaraldehyde). **(B)** Measurements of the elongation index for living RBCs vs. populations treated with 0.0001, 0.001, 0.01, 0.1, and 1% glutaraldehyde. **(C)** Histograms for the determination of the elastic modules of living RBCs vs. their treatment with 0.001 and 0.0025% glutaraldehyde. Please note that the histograms partly overlap, e.g., the brown appearing columns represent the overlap of the orange and gray columns. The inset are illustrate the AFM measurements. The images show a RBC fixed with 0.0025% glutaraldehyde. The color code of the left image depicts the height/thickness of the cell, while the right image represents slopes of force-displacement curves. Scale bars indicate 5 μm. Please note that the indicated glutaraldehyde concentrations in all panels are batch-specific and not of general validity.

Basing on the assumption that fixed cells may be conserved for a long time (ideally months or even years) with no morphological changes or lysis of the cells, we investigated the shelf life by treating samples with various glutaraldehyde concentrations kept at 4°C for 6 days. Every 2 days the supernatant sample was prepared to measure the free hemoglobin content in the suspensions. It correlates directly to the amount of lysed cells in the samples. Figure 2A reveals glutaraldehyde’s seemingly digital (all or none) fixation property. Percentages above 0.01% showed almost no hemoglobin in the supernatant. At lower concentrations, increase in glutaraldehyde percentage increase the hemolysis rate compared to unfixed control cells until glutaraldehyde reaches a concentration were RBCs are fixed.

To probe the stiffness of glutaraldehyde-treated RBCs we used an ektacytometer, LoRRca, a routine tool to investigate hemolytic anemias (Zaninoni et al., 2018). Figure 2B reveals that very low concentrations of glutaraldehyde (here: 0.0001%) resemble the mechanical behavior of living cells, while concentrations above 0.01% glutaraldehyde are (in agreement with Figure 2A) indistinguishable in their elongation index plot. Interestingly, 0.001% glutaraldehyde shows indeed a curve/behavior in between the two extreme conditions. Using atomic force spectroscopy, we had a closer look in the latter range and compared living RBCs with 0.001 and 0.0025% glutaraldehyde treated RBCs (Figure 2C). On the one hand this plot shows that indeed the cell populations are shifted in their elastic module in dependence of the glutaraldehyde concentration. On the other hand, one can also notice a wide and overlapping spread of the elastic modulus (please note the logarithmic scale).

### Correction for Osmolality

**FIGURE 3:**
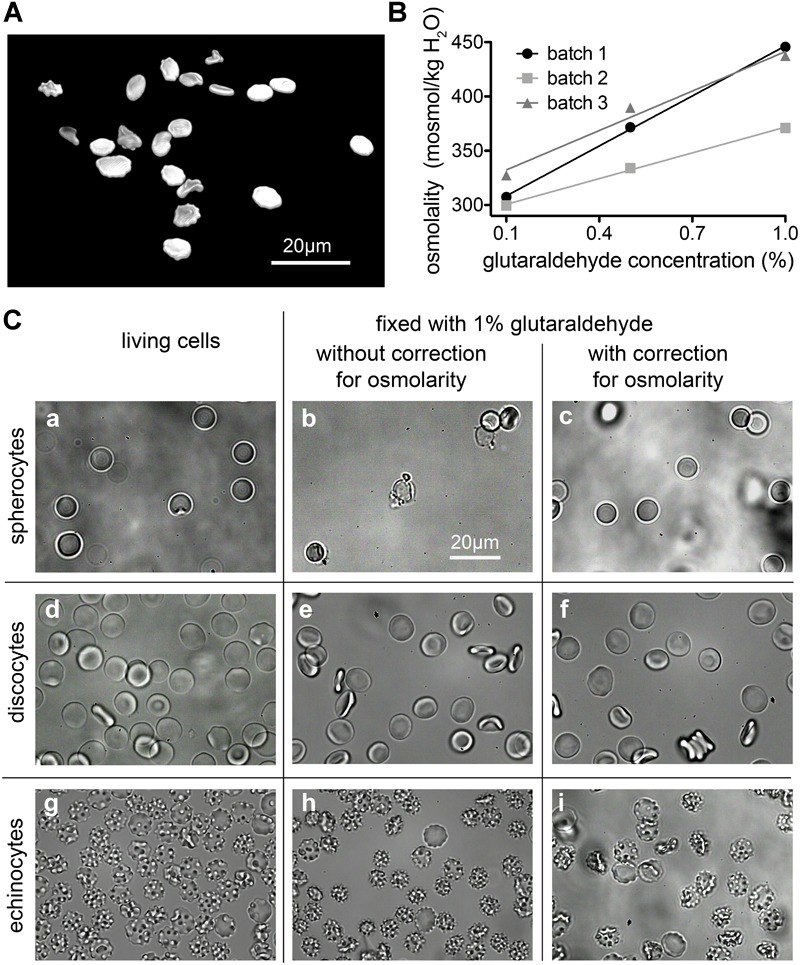
Osmolality changes induced by glutaraldehyde. **(A)** Patient sample (pyruvate kinase deficiency) fixed in 1% glutaraldehyde without correction for osmolarity (495 mosmol/kg H_2_O). The resulting dehydrated cells have a flat aspect and borders and are spiky like in echinocytes. **(B)** Relation between nominal glutaraldehyde concentration and osmolality in PBS for three different batches of glutaraldehyde. **(C)** RBCs shapes are drastically influenced by osmolality. Spherocytes can only preserve their shape for about 127 to 145 mosmol/kg H_2_O **(a–c)**. Although for the uncorrected 1% glutaraldehyde fixation **(b)**, one would expect a shrinkage of the cell, we observed a high percentage of lysis with only a few cells left. Discocytes **(d–f)** can preserve their shape for osmolality values between 210 and 380 mosmol/kg H_2_O. 290 mosmol/kg H_2_O is the aimed osmolality for most mediums and it was the osmolality value that fixation solutions were corrected to be for this study. For the echinocytes **(g–i)** a more pronounced shrinkage can be seen in uncorrected 1% glutaraldehyde solution **(h)**. The detailed osmolalities are (in mosmol/kg H_2_O) for **(a)**: 131; **(b)**: 214; **(c)**: 122; **(d)**: 291; **(e)**: 389; **(f)**: 292; **(g)**: 802; **(h)**: 877; **(i)**: 774. Please note that the indicated glutaraldehyde concentrations in all panels are batch-specific and not of general validity.

Changes in the osmotic pressure result in highly deformed shapes, which lead to the formation of flattened discocytes with a clear shrunk appearance. We came across artificially deformed cells when we fixed RBCs of a patient with pyruvate kinase deficiency (Figure 3A). The shrinkage of the RBCs is proportional to the concentration of the glutaraldehyde (Figure 3B,C) and although the osmolality increase of glutaraldehyde solution scales linearly with its concentration, there are severe differences between glutaraldehyde batches as outlined in Figure 3B. While the change in osmolality might not be relevant in certain cell types in terms of morphology (Machado, 1967; Bone and Denton, 1971; Rasmussen, 1974), for RBCs we observed that the osmolality is essential for shape preservation (Figure 3C).

We are aware of the fact that glutaraldehyde may not be osmotically active. It is an uncharged, rather hydrophobic molecule that crosses the membrane, as it clearly reacts with intracellular proteins with a fast kinetics. Whether an osmotic effect could be present and relevant depends on the differential permeability of the membrane to glutaraldehyde itself and to water. Glutaraldehyde appears to act rapidly and to fix RBCs within 1 s (Sutera and Mehrjardi, 1975). Water fluxes across the RBC membrane are also fast owing to the presence of aquaporin. So the two permeabilities may be seen as to be approximately equivalent. Yet it is unknown if, and to what extent these parameters change in the time frame of glutaraldehyde reacting with the membrane, for instance affecting its own permeability coefficient and/or that of aquaporin, not to mention all the other protein-mediated transport systems of the RBC membrane. In the literature it is also argued that the buffer osmolality plays a more important role than the osmolality increase caused by glutaraldehyde addition (Barnard, 1976; González-Aguilar, 1982). Despite of all this, we have nonetheless observed that osmolality compensation by dilution of the buffer is essential for the preservation of shapes within the spherocyte-discocyte-echinocyte range, especially when glutaraldehyde is used in the high concentration range.

For discocytes, as they are the most stable shape for the cells, the range of tolerable osmolality is wide. Discocytes can preserve their shape for osmolality values between 210 and 380 mosmol/kg H_2_O. Ideally 290 mosmol/kg H_2_O is the aimed osmolality for most media, and it was also the osmolality value that fixation solutions were corrected for within this study. However, rare cell shapes are a lot more sensitive to osmolality changes. For example; spherocytes can only preserve their shape for about 127 to 145 mosmol/kg H_2_O, which is a much narrower range.

### Monomers and Polymers

**FIGURE 4:**
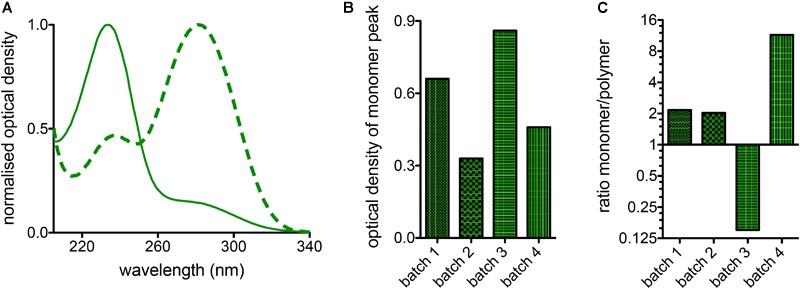
Batch variations of glutaraldehyde. **(A)** Normalized absorption spectra of two different batches of glutaraldehyde in distilled water. The solid line shows predominantly the monomere peak, whereas the highest band of the dashed line represents the polymers. **(B)** Comparison of the monomer absorption peak for 4 batches of glutaraldehyde with nominally the same concentration of 1% in distilled water. **(C)** Ratio between monomers and polymers derived from 1% glutaraldehyde solutions in distilled water for 4 different batches of glutaraldehyde.

In a glutaraldehyde solution monomers and polymers coexist (compare Figure 1). Both monomers and polymers have different “fixation properties.” In particular, the higher efficiency in crosslinking due to monomers or polymers is discussed controversially (Richards and Knowles, 1968; Hardy et al., 1969; Robertson and Schultz, 1970; Gillett and Gull, 1972). While preparation/purification to yield pure monomeric and polymeric solutions requires a certain preparation effort, the analysis of differences or variations of cell ultrastructure in electron microscopy (Bessis, 1974) goes far beyond the frame of this report. However, it is of interest to evaluate the particular composition of glutaraldehyde, which can be measured by UV-absorption spectroscopy as indicated in Figure 4A depicting the large variety of glutaraldehyde solutions used in our laboratories. The ratio of absorbance between 280 nm (monomer, Figure 4B) and 235 nm (polymer) can be taken as the monomer-polymer ratio (Figure 4C). Our results show indeed that glutaraldehyde polymerization can vary between batches. Change over time, in particular when stored at room temperature is the most likely cause for the differing ratios. In addition and independent of the polymerization, we found variations in the concentration of different glutaraldehyde batches for nominal equal concentrations as indicated by the optical density of the monomer peak depicted in Figure 4B. Therefore, it is important to document both, the monomer to polymer ratio of glutaraldehyde as well as the peak of the monomer concentration as a reference.

### Handling Pitfalls

**FIGURE 5:**
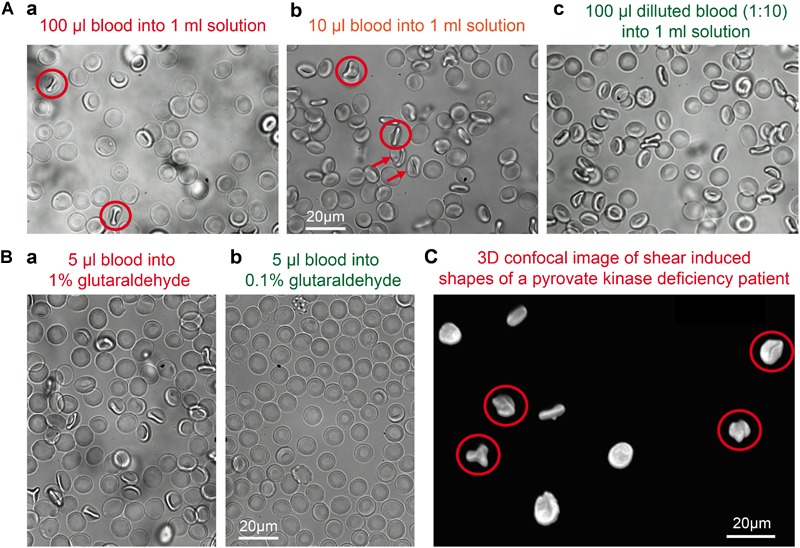
Shear induced artifacts during glutaraldehyde fixation. **(A)** Representative fixation examples in 1% glutaraldehyde solution in dependence of the sample preparation. **(a)** 100 μl of whole blood pipetted into a 1% glutaraldehyde solution; **(b)** 10 μl of whole blood pipetted into a 1% glutaraldehyde solution; **(c)** 100 μl of a 1:10 diluted blood suspension pipetted into a 1% glutaraldehyde solution. Circles indicate knizocytes and arrows otherwise deformed RBCs. **(B)** Representative fixation examples of 5 μl whole blood in **(a)** 1% glutaraldehyde and **(b)** 0.1% glutaraldehyde. **(C)** Patient sample (pyruvate kinase deficiency) fixed in 1% glutaraldehyde with cells showing shear induced artifacts as the knizocytes marked with circles. Please note that the indicated glutaraldehyde concentrations in all panels are batch-specific and not of general validity.

When RBCs are pipetted into a glutaraldehyde solution they are believed to be fixed within 1 s (Sutera and Mehrjardi, 1975). This means that if the pipetting creates a considerable flow (compare also Wiegmann et al., 2017), the cellular shape adaptation to flow (Lanotte et al., 2016; Quint et al., 2017) is also conserved, which is a deviation from RBC shape in stasis (Figure 5) and must be regarded as an artifact. The cells marked by red circles in Figure 5 include knizocytes (trilobes), a clear indication for flow induced cell shape changes (Lanotte et al., 2016). Gentle pipetting is compulsory when fixing with 1% or higher glutaraldehyde concentrations. In addition a pre-dilution in saline solution of the blood sample to a hematocrit of 5% or lower proved to be helpful, due to a decrease in the viscosity of the suspension (Eckmann et al., 2000) and thus a decrease in shear stress on the cells.

In microfluidics, glutaraldehyde treated cells are often used to test deformability-based cell sorting devices, as well as to study flow of non-deformable particles (Holmes et al., 2014; Tomaiuolo et al., 2015). One common problem that often occurs while working with fixed cells is the increased sedimentation rate due to higher density of cells. This disadvantage can be improved upon by using a lower glutaraldehyde concentration for fixation. The density of cells that have been fixed with 1% was measured to be 1.21 g/ml whereas the density for 0.1% fixed cells is 1.18 g/ml, while the average cell density of living cells is 1.10 g/ml. We considered two possible reasons that could explain the increase in density of cells post-fixation: either the cross-linking caused a decrease in the cell volume (cellular dehydration), or the binding of the glutaraldehyde molecules on the cell increased the mass of the cell. Since glutaraldehyde has molar density of 0.933 g/ml (Zang et al., 2016), which is lower than both surrounding medium and the healthy cell density (1.1 g/ml), the addition of glutaraldehyde molecules to the cell membrane is unlikely to increase the density of the cell. From the density measurements we can calculate that 1% glutaraldehyde fixation results in approximately 9% volume decrease, whereas 0.1% fixation results in approximately 7%. A possibility to counterbalance the increase in density is the adaptation of the density of the suspension, e.g., by using OptiPrep (Stemcell Technologies, Vancouver, BC, Canada).

### Glutaraldehyde Induced Fluorescence and Staining of Fixed Cells

Staining of fixed RBCs requires to consider both the possible interference of hemoglobin with fluorescent dyes (Kaestner et al., 2006) and the fluorescence induced by glutaraldehyde. Although the autofluorescence of the glutaraldehyde itself is negligible it forms fluorescent entities upon binding to peptides and proteins (Lee et al., 2013).

**FIGURE 6:**
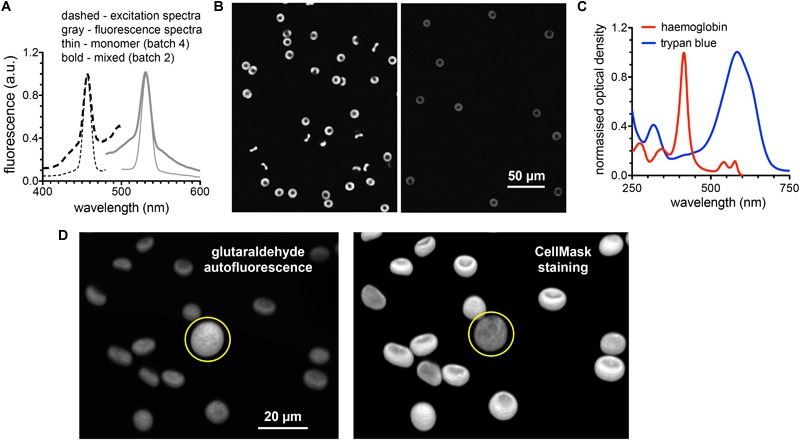
Fluorescence induced by glutaraldehyde fixation. **(A)** Normalized excitation and fluorescence spectra of a RBC suspension fixed with 1% glutaraldehyde. **(B)** Confocal images of RBC fixed with 1% glutaraldehyde before (left image) and after (right image) suspending the cells in trypan blue solution. **(C)** Normalized absorption spectra of hemoglobin and trypan blue to spectrally judge fluorescence quenching. **(D)** 3D images of RBCs fixed with 1% glutaraldehyde. Left image shows the autofluorescence based on the excitation wavelength of 561 nm (no excitation of CellMask). Right image shows the CellMask fluorescence of the same cells excited with a wavelength of 647 nm (only residual glutaraldehyde induced fluorescence). The yellow circle marks a white blood cell. Please note that the indicated glutaraldehyde concentrations in all panels are batch-specific and not of general validity.

To judge the fluorescence induced by glutaraldehyde, the excitation and emission spectra of the glutaraldehyde fixed RBCs is presented in Figure 6A. We present spectra of cell suspensions fixed with 2 different batches of glutaraldehyde. For the almost exclusive monomeric glutaraldehyde (batch 4 in Figure 4C), the induced fluorescence contains distinct narrow bands (thin lines in Figure 6A), whereas the mixture of monomers and polymers (batch 2 in Figure 4C) gives in addition to the monomer band a wide band fluorescence background (bold lines in Figure 6A), exceeding the range of the spectra measured. This demonstrates that the induced fluorescence can be a problem in immunofluorescence staining (see also below), also considering the wide spectral range of the induced fluorescence, in particular in the presence of glutaraldehyde polymers. Especially when quantitative fluorescence intensity measurements are required, it is crucial to take the glutaraldehyde induced fluorescence into account. Therefore, we present a previously described method to quench the fluorescence by addition of trypan blue (Loike and Silverstein, 1983). Confocal sections of glutaraldehyde fixed RBCs (excitation at 488 nm) in the absence and presence of trypan blue are exemplified in Figure 6B. Although the fluorescence signal is reduced, it could not be completely excluded. To evaluate the putative effect of trypan blue, the absorption spectrum is depicted in Figure 6C. Additionally, the absorption spectrum of hemoglobin is plotted in Figure 6C.

To evaluate the putative use of the glutaraldehyde induced fluorescence for RBC imaging we present in Figure 6D 3D-images of glutaraldehyde fixed and CellMask stained cells. While the left image in Figure 6D predominantly presents glutaraldehyde induced fluorescence, the right image depicts mainly CellMask fluorescence. To highlight the special properties of RBCs we choose an image that contains by chance a white blood cell (marked with a yellow circle in Figure 6D).

**FIGURE 7:**
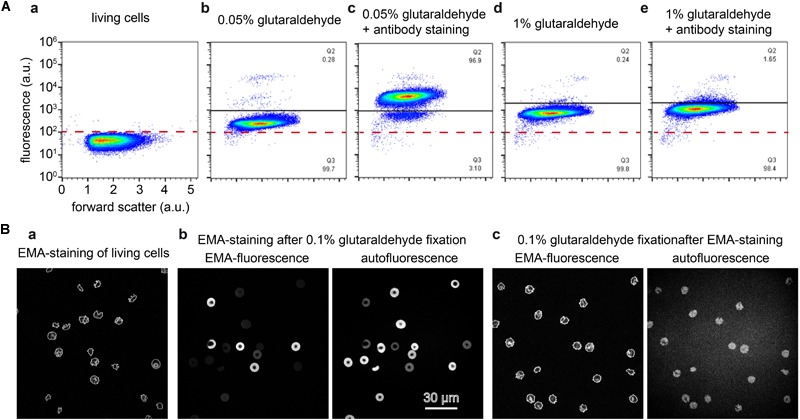
Staining of glutaraldehyde fixed samples. **(A)** Flow cytometric analysis of cord RBCs for the detection of cytosolic fetal hemoglobin F. Panels **(a–e)** are plotted at the same scale as annotated in panel **(a)**. **(a)**: unstained control RBCs; **(b)**: cord blood fixed with 0.05% glutaraldehyde; **(c)** cord blood fixed with 0.05% glutaraldehyde and consecutively stained with an antibody against hemoglobin F; **(d)** cord blood fixed with 1% glutaraldehyde; **(e)**: cord blood fixed with 1% glutaraldehyde and consecutively stained with an antibody against hemoglobin F. **(B)** Eosin-5′-maleimide (EMA) staining of RBCs. **(a)**: EMA staining of living cells induces cell shape changes. **(b)**: EMA staining of 0.1% glutaraldehyde fixed RBCs, the left image is excited at 488 nm, the excitation peak of EMA with only residual glutaraldehyde induced fluorescence. The right image is excited at 647 nm where no EMA fluorescence is induced but the residual glutaraldehyde induced fluorescence is sufficient to image RBCs. Both images reveal a very heterogeneous fluorescence, whereas the fluorescence intensity is inverted, i.e., cells with a high EMA fluorescence show a low glutaraldehyde induced fluorescence and vice versa. **(c)**: 0.1% glutaraldehyde fixation of EMA stained cells, the images are recorded under the same conditions as in **(b)**. The left image depicts a similar pattern as the living cells presented in **(a)**, while the right image shows the glutaraldehyde induced fluorescence. Please note that the indicated glutaraldehyde concentrations in all panels are batch-specific and not of general validity.

To further illustrate the scenario of interaction between glutaraldehyde and the staining of proteins of interest we present two prominent examples of (i) a cytosolic protein and (ii) a membrane protein. (i): We performed flow cytometric measurements of living RBCs and fixed cells with different concentrations of glutaraldehyde with and without additional staining of the (cytosolic) hemoglobin F (Figure 7A). Induced fluorescence is high at 1% glutaraldehyde but lower at decreased concentrations. Furthermore, flow cytometry data reveal higher fluorescence for samples kept in glutaraldehyde for longer time (several hours, data not shown). Moreover, cells showed different fluorescence intensities within the same sample, underlining different glutaraldehyde binding amounts between individual cells. This might reflect cell protein content, which varies between cells, e.g., in dependence of RBC age (Kaestner and Minetti, 2017). Additionally, we observe the effect that long staining with high (1%) concentrations of glutaraldehyde prevents binding of the antibodies (Figure 7Aa–e). However, hemoglobin F antibodies (FITC) can distinctively be identified in 0.05% glutaraldehyde fixation. (ii): We imaged RBCs stained with EMA, which binds to the amino group of Lys-430 on RBCs membrane Band 3 protein and it is commonly used to estimate Band 3 protein abundance. Figure 7B compares images of living cells (Figure 7Ba), EMA staining after 0.1% glutaraldehyde fixation (Figure 7Bb) and fixation with 0.1% glutaraldehyde after EMA staining (Figure 7Bc).

**FIGURE 8:**
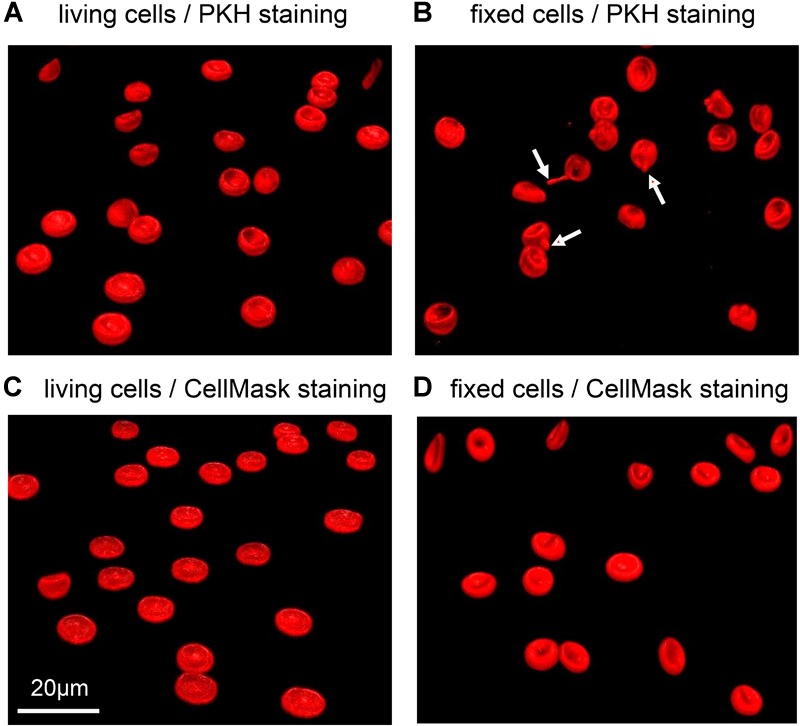
Representative confocal 3D-images of membrane stained RBCs. **(A)** and **(B)** show RBCs stained with PKH67, while **(C)** and **(D)** are stained with CellMask deep red. **(A)** and **(C)** depict living RBCs, while **(B)** and **(D)** contain cells fixed in 1% glutaraldehyde. The red arrows point to the filaments formed in fixed RBCs stained with PKH67. Please note that the indicated glutaraldehyde concentration in panels **(B)** and **(D)** are batch-specific and not of general validity.

Also the choice of the particular dye might be influenced by the glutaraldehyde. This statement goes beyond the obvious spectral selection presented above. For illustration we tested two types of cell membrane fluorescent staining dyes both on living and fixed cells: PKH dyes proved to be reliable for RBC *in vivo* staining for several weeks (Wang et al., 2013); CellMask is a popular dye to stain RBC *in vitro* (Flormann et al., 2017). While for living cells we observed a homogeneous membrane staining, the dyes did not always show to be ideal on fixed cells. PKH26 as well as PKH67 in particular resulted in the formation of visible “filaments” forming at the cell membrane (Figure 8). For CellMask we occasionally noticed the formation of small accumulations of dye in certain fixed samples, when staining at low concentrations. Such an inconsistent effect could be due to the monomer/polymer ratio of the glutaraldehyde affecting the crosslinking of cells.

### Special Emphasis on Sickle Cell Disease

**FIGURE 9:**
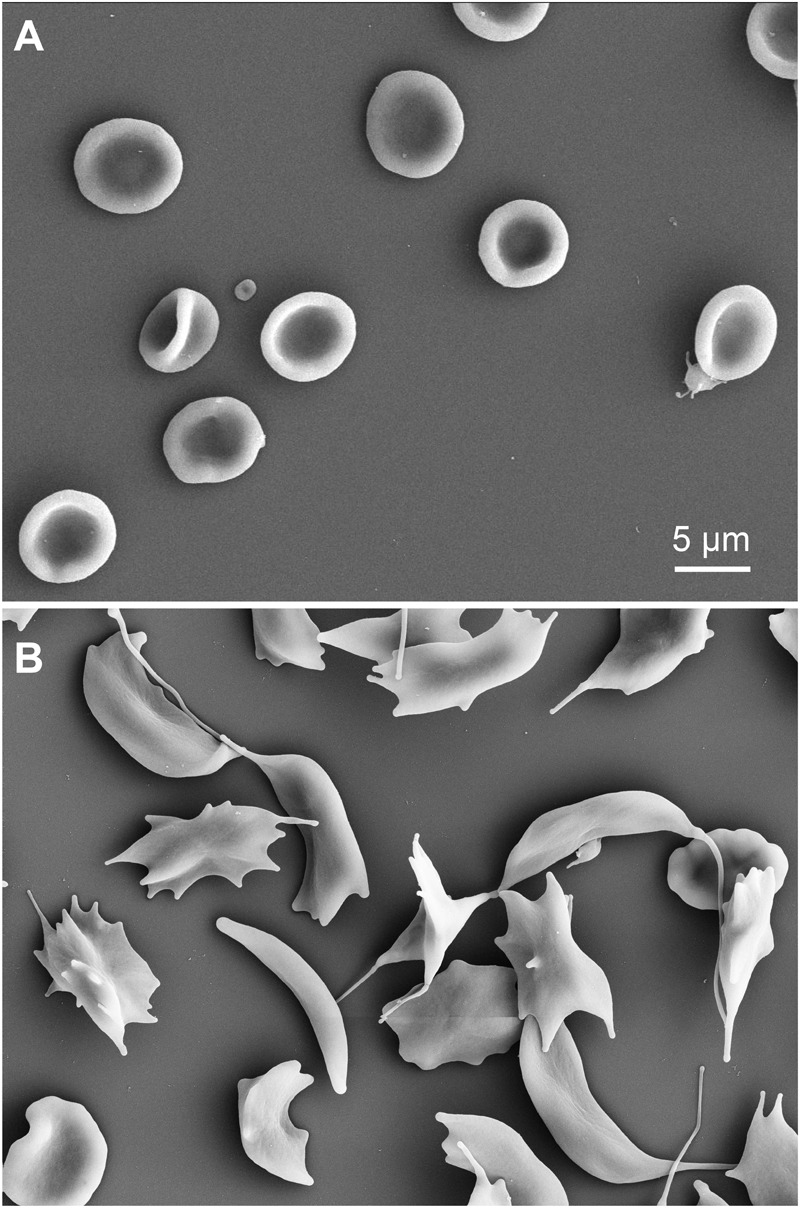
Scanning electron microscopy images of RBCs of a sickle cell disease patient. **(A)** oxygenated cells fixed with 1% glutaraldehyde. **(B)** deoxygenated RBCs (8 h in a hypoxic glove box with 0.2% PO_2_) of the same sickle cell disease patient as in **(A)** also fixed with 1% glutaraldehyde. Please note that the indicated glutaraldehyde concentrations in all panels are batch-specific and not of general validity.

Since glutaraldehyde consumes oxygen during its reaction with compounds (Johnson, 1987), we investigated if a glutaraldehyde induced deoxygenation could cause hemoglobin crystallization in sickle cell disease patients, transforming discocytes containing oxygenated hemoglobin into sickle cells. To this end we fixed oxygenated and deoxygenated RBCs of a sickle cell disease patient with 1% of glutaraldehyde and performed scanning electron microscopy (SEM). The resulting SEM images are presented in Figure 9, clearly showing discocytes in oxygenated cells (Figure 9A) and sickled cells in deoxygenated RBCs (Figure 9B) of the same patient.

## Discussion

### Fixation vs. Modulation of Stiffness

Previous literature gives different meanings to the concept of “fixation”: conservation of cells (Nowakowski and Luckham, 2002; Faivre et al., 2006; Karon et al., 2012) and regulation of cellular rigidity (Guo et al., 2014; Tong and Caldwell, 1995). This study emphasizes the subtle difference between the two concepts as presented in Figure 2A,B. Fixation of cells with glutaraldehyde and increase in cellular stiffness are two different pairs of shoes. Fixation can be thought of as a binary state (all or none), whereas rigidity can be roughly regulated at low glutaraldehyde concentrations, as shown by ektacytometry (Figure 2B) and atomic force spectroscopy (Figure 2C). However, for the latter application one should avoid the term “fixation,” because instead of being fixed, cells are rather more fragile (Figure 2A). Please note that the concentrations of glutaraldehyde given in Figure 2 and in the results sections can’t be regarded as general numbers, because the batch to batch differences (Figure 4 and discussion below), probably highly influenced by the ratio of monomers and polymers, have a tremendous influence on the fixation properties. Furthermore, glutaraldehyde appears to have a toxic effect on the cells for 0.0001 and 0.001% concentrations (Figure 2A), since they exhibited more lysis than the control case (living cells). The toxicity effect also can explain the increase of lysis from a 0.0001 to 0.001%.

### Correction for Osmolality

Unlike some other types of cells, RBC morphology is highly sensitive to several parameters, e.g., mechanical stress, pH changes, addition of chemical agents and osmolality differences. Because of such changes, RBC shapes are thoroughly studied in blood diseases, where genetic mutations and/or impaired cell functions affect cell shapes (Ponder, 1948; Delaunay, 2004; Diez-Silva et al., 2010; Fermo et al., 2017). This implies that morphological analysis must be performed on reliable samples. Cell fixation is an ideal step when working with rare anemias, where patients’ samples often need to be investigated in specialized laboratories and may be stored before the analysis can be performed (Figure 3B). It is well known that RBC shapes are sensitive to osmolality (Reinhart et al., 2015). Adding 1% glutaraldehyde to a given solution increases the osmolality by approximately 100 mosmol/kg H_2_O, which results for physiological solutions in a relevant osmolality change of approximately 30%. Even if fixation occurs within seconds, the osmotic pressure difference allows water to flow in or out of the cell, affecting the shape that will be fixed. Such a water flow is also observed at higher glutaraldehyde concentrations, where the increase in osmolality is proportionally higher while fixation occurs faster (data not shown). Our results show that each shape has a different tolerance to osmolality, spherocytes being the most fragile and unstable shape (Figure 3A). Therefore the evaluation of spherocytes underlines the importance of osmolality correction in the fixative. If the osmolality is particularly high, discocytes appear flattened and borders slightly spiky, resembling echinocytes. Cells do not fully deform into echinocytes probably because of the simultaneous crosslinking of glutaraldehyde while dehydration occurs. In the case of discocytes, a judgment on fixed shape quality becomes generally more difficult because the shape change is less evident and often subjective to the observer. Therefore we see a need for automated (unbiased) cell shape analysis algorithms. A visual inspection is not enough for comparison with living cells, even in 3D imaging. We can, however, mention that cells fixed in 1% corrected glutaraldehyde resemble cells fixed in 0.1% glutaraldehyde, where the osmotic imbalance after addition of glutaraldehyde to the buffer is minimal (10 mosmol/kg H_2_O). Therefore we suggest to verify the osmolality of the fixative solution before fixation of any samples, particularly when dealing with patients that display shape deformations, e.g., hereditary spherocytosis, sickle cell disease or pyruvate kinase deficiency. Such diseased RBCs might be more severly affected by environmental changes, like osmolarity, than healthy discocytes.

### Monomers and Polymers

Glutaraldehyde solutions are mixtures of monomers and polymers as outlined in Figure 4. We did not investigate the advantages and disadvantages of monomers and polymers for the fixation of RBCs but this topic was previously discussed controversially (Richards and Knowles, 1968; Hardy et al., 1969; Robertson and Schultz, 1970). However, it is evident that the ratio of monomers and polymers can vary between different glutaraldehyde batches (Figure 4C), but the ratio can easily be determined by UV-absorption spectroscopy. Previous studies (Gillett and Gull, 1972; Rasmussen and Albrechtsen, 1974; Prentø, 1995) demonstrated that the storage temperature of glutaraldehyde is among the most important parameters for a stable preservation of the stock solution. Rasmussen (1974) has tested different storage temperatures, concluding that glutaraldehyde is most stable at -20°C. The higher the temperature, the faster the increase in polymer formation. However, we point out that the uncontrolled formation of polymers in the stock solution will affect the impact of glutaraldehyde on osmolality, therefore making it necessary to check the osmolality increase for every prepared solution before applying osmolality correction.

### Handling Pitfalls

As stated before, RBC morphology is affected by mechanical stress, e.g., flow, to which RBCs adapt by changing their shapes into hydrodynamic ones. Pipetting results in shear of RBCs, which leads to the formation and fixation of flow shapes like knizocytes (also referred to as trilobes) (Lanotte et al., 2016), known to form at high shear rates (Figure 5). This highlights that the speed of fixation is extremely fast, knowing that RBCs in flow relax to the static shape in between 100 ms (Amirouche et al., 2017) and 1 s (Braunmüller et al., 2011). We observed knizocytes when fixing with 1% glutaraldehyde, but not when fixing with lower concentrations, hinting to a slower fixation for lower concentrations. Such distortions of the regular cell shape can cause confusion when diseased RBCs are under investigation (Figure 5C). To avoid the presence of flow induced shapes, we found it is sufficient to dilute the blood sample to hematocrits of 5% in a solution of lower viscosity, e.g., PBS.

### Staining of Fixed Cells

The fluorescence induced by glutaraldehyde is significant and covers a wide range of wavelengths (Figure 6A, 7A), which can be essential for the fluorescence measurement as exemplified by the measurement of hemoglobin F in cord blood RBCs using flow cytometry (Figure 7A) or by staining for Band 3 protein with EMA (Figure 7B). Although glutaraldehyde induced fluorescence can in principle be used to image RBCs (Figure 6B,D), the 3D-reconstruction reveals that fluorescence intensity is limited and does not reach dedicated staining such as CellMask staining (Figure 6D).

However, for fluorescent staining of membrane proteins that do not require permeabilization of the RBCs we recommend to first perform the membrane staining followed by the fixation. Already the staining of Band 3 protein with EMA showed severe inconsistencies that are likely to result from the fact that glutaraldehyde also binds to amino groups, i.e., EMA needs to compete with glutaraldehyde for putative binding sites (compare Figure 7Bb). If this is already evident for the highly abundant Band 3 protein, we expect even more severe effects for less abundant proteins and their detection using antibodies.

According to the application needed, we recommend to fix cells shortly if the autofluorescence signal is not desirable, meaning few minutes, using low concentrations to avoid higher autofluorescence than the signal coming from a specific staining. Using monomeric glutaraldehyde, the autofluorescence induced is in a narrower spectral range compared to polymeric glutaraldehyde (Figure 6B). This results in a several fold reduced autofluorescence at the popular laser line 488 nm, which is also advantageous in respect to the absorption properties of both hemoglobin and trypan blue (Figure 6C). Quenching tests revealed trypan blue to be efficient, however, not completely eliminating the signal (Figure 6C,D).

Membrane staining showed to be different between living and fixed cells (Figure 8). While CellMask is toxic at high concentrations on living cells, PKH dyes might not provide an appropriate staining on fixed cells, even at low glutaraldehyde concentrations. PKH consists of a fluorescent dye incorporated in a long aliphatic chain that inserts into membrane lipids. Due to the presence of glutaraldehyde crosslinking the membrane proteins, it might be that PKH cannot be appropriately inserted into the membrane, giving the formation of protrusions that affect the quality of the staining (Figure 8B). CellMask is an amphipathic molecule linked to a charged dye that can be used both for the staining of living and fixed cells and resulted in fact in a more efficient labeling of fixed cells with any tested concentration of glutaraldehyde.

### Sickle Cells

Generally, the effect of O_2_ consumption during glutaraldehyde fixation is clearly visible in RBCs suspension, which upon fixation become darker, as a consequence of methemoglobin formation (Guillochon et al., 1986). However, up to 1% of glutaraldehyde based fixation of sickle cells we could not detect any sickling as outlined in Figure 9A and therefore we like to exclude hemoglobin crystallization due to deoxygenation.

## Conclusion

In this section we will provide general recommendations to use glutaraldehyde to fix or rigidify RBCs. Due to glutaraldehyde variations between commercial providers, batch to batch variations and conversions of glutaraldehyde during storage time, these recommendations can hardly provide particular numbers neither for the minimal glutaraldehyde concentration to fix or rigidify the cells nor for the exact osmolality compensation. Instead we recommend procedures to consider, with the aim to ease glutaraldehyde use.

(i) We recommend to store glutaraldehyde at -20°C in aliquots to avoid unnecessary thawing of the whole bottle for each use. Use these aliquots to prepare fresh solutions before treating the cells. This allows a maximal consistency within a series of measurements in a particular laboratory over time.
(ii) The glutaraldehyde concentration to be used depends on the purpose und needs to be determined for the particular application and the particular glutaraldehyde batch. If fixation is required, a quick control can be done by washing the cells in distilled water prior to experiments. Fully fixed, fully non-deformable cells, will not lyse or have any morphological changes (test the supernatant for hemoglobin). In general, concentrations between 0.05 and 0.1%, fix cells but do not require osmolality compensation, limit the induced fluorescence as well as the increase in the density of cells. However, attention should be given, because fixation is not immediate but take longer time. In contrast, concentrations of glutaraldehyde around 1% require an osmotic compensation (see below) and if induced fluorescence is not a hindering aspect, 1% glutaraldehyde provides a sound fixation and is ideal for electron microscopy applications, where often even higher percentages of glutaraldehyde are used as these methods are physically more abrasive to the cells compared to optical imaging. Additionally, we like to mention the opportunity to set a fixation at a rather low glutaraldehyde concentration, e.g., 0.05 – 0.1% and increase the concentration in a second step to 1% to avoid insufficient glutaraldehyde content.
(iii) We suggest to measure the osmolality of the glutaraldehyde containing solution and correct it by diluting the suspension buffer when fixing with 0.5% or higher concentrations of glutaraldehyde.
(iv) It is recommended to check the monomer/polymer ratio of the glutaraldehyde using a spectrometer and provide this ratio in the publications as a way to help interlaboratory comparability.
(v) It is necessary to consider the appropriate staining dye and its concentration in relation to the induced autofluorescence. If cell shapes are considered, the autofluorescence is rather advantageous. Staining based on the recognition of protein structures (antibodies) or relying on the access to intracellular structures have to be handled with care and should pre-tested on glutaraldehyde treated positive controls to show the feasibility. Trypan blue can be used to quench the autofluorescence. Staining of membrane proteins (e.g., using dyes or cluster of differentiation antibodies) should be performed prior to fixation.

## Ethics Statement

The procedure is approved by the local ethics committee (Approval No. 51/18) and was performed in accordance with the Helsinki international ethical standards on human experimentation.

## Author Contributions

All authors listed have made a substantial, direct and intellectual contribution to the work, and approved it for publication.

## Conflict of Interest Statement

The authors declare that the research was conducted in the absence of any commercial or financial relationships that could be construed as a potential conflict of interest.
